# Editorial: Purinergic pharmacology, Volume II

**DOI:** 10.3389/fphar.2023.1200187

**Published:** 2023-05-16

**Authors:** Francisco Ciruela, Kenneth A. Jacobson

**Affiliations:** ^1^ Pharmacology Unit, Department of Pathology and Experimental Therapeutics, School of Medicine and Health Sciences, Institute of Neurosciences, University of Barcelona, Llobregat, Spain; ^2^ Neuropharmacology and Pain Group, Neuroscience Program, Bellvitge Biomedical Research Institute, Llobregat, Spain; ^3^ Molecular Recognition Section, Laboratory of Bioorganic Chemistry, National Institute of Diabetes and Digestive and Kidney Diseases, National Institutes of Health, Bethesda, MD, United States

**Keywords:** ATP, G protein-coupled purinergic receptors, adenosine, purinergic signalling, adenosine transport, purinergic pathophysiology, ligand-gated ion channels, guanosine

Extracellular purine nucleotides and nucleosides serve as crucial signalling molecules, acting as neurotransmitters and neuromodulators. Tightly regulated extracellular levels of adenosine 5′-triphosphate (ATP) and adenosine, which are controlled by various enzymes and transporters, activate a variety of purinergic receptors. These receptors, which appear early in evolution, are among the most abundant receptors in living organisms and regulate numerous physiological processes, making them attractive therapeutic targets for a wide range of diseases. While P1 (adenosine) receptors are selective for adenosine, the breakdown product of ATP, P2 receptors respond to purine and pyrimidine nucleotides. Importantly, purinergic receptors, including both G protein-coupled receptors (ARs and P2YRs) and ligand-gated ion channel receptors (P2XRs), are involved in a multitude of neuronal and non-neuronal mechanisms, such as pain, immune responses, exocrine and endocrine secretion, platelet aggregation, endothelium-mediated vasodilatation, and inflammation. However, since purinergic receptors are widely distributed throughout the body, it is challenging to develop drugs that selectively target specific receptor subtypes without causing unwanted side effects. Additionally, extracellular levels of purines and pyrimidines can also vary greatly, leading to the simultaneous activation of different purinergic receptors in response to oscillating concentrations of endogenous purines. Consequently, through these different subtypes of P1 and P2 receptors, cells integrate extracellular purine responses, harmonising short- and long-term purinergic signalling. Therefore, the selectivity of drugs is a crucial goal in the field of purinergic pharmacology. For decades, medicinal chemists have been developing potent and selective synthetic agonists and antagonists for purinergic receptors, as well as allosteric modulators that allow for event-responsive and temporally specific manipulation of the endogenous purinergic system. Additionally, modulation of the metabolism and uptake of extracellular purine nucleotides and nucleosides can also regulate purinergic processes. Overall, the field of purinergic pharmacology is rapidly expanding and presents exciting opportunities for pharmacotherapeutic development.

In this Research Topic that follows an earlier volume on the same Research Topic (Ciruela and Jacobson), an overview of purinergic pharmacology is provided through 13 articles written by 82 authors. This comprehensive compilation contains 1 minireview, 1 review, 1 brief research report, and 10 original research articles. The minireview provides an update on the recent development of allosteric modulators for adenosine receptors and their therapeutic applications (Pasquini et al.). The authors highlight that allosteric modulators of ARs may evolve into valuable pharmacological tools that can overcome the limitations of orthosteric ligands. Thus, while several allosteric modulators have been identified for A_1_R and A_3_R, with some promising results in preclinical settings, the discovery of allosteric modulators for A_2_R and A_2B_R has been less successful, although current findings are still encouraging. Despite the promising results, no AR allosteric modulator has yet advanced to clinical trials, highlighting the considerable challenges involved in the discovery and development of this class of compounds. Adenosine signalling is known to be upregulated during periods of restricted oxygen availability, such as those experienced during ischemic events or inflammatory conditions. Yuan et al. elegantly review the molecular connection between netrin-1 and A_2B_R and summarise relevant research on their interaction within the context of tissue inflammation (Yuan et al.). It is suggested that netrin-1 may serve as an endogenous anti-inflammatory agent during organ injury by enhancing the adenosine-A_2B_R axis, which has anti-inflammatory properties.

The Research Topic contains a series of original research articles covering important aspects of purinergic pharmacology. A brief research report article shows the use of machine learning models to find ligands for the different AR subtypes (Puhl et al.). The authors have made an interesting discovery by identifying three new modulators for ARs (crisaborole, febusostat, and paroxetine) that are structurally different from previously known compounds targeting ARs. These medications, in some cases suggesting noncanonical interaction with ARs, could potentially be repurposed for adenosine-related diseases. Five articles revolve around P2XR, with particular attention paid to P2X7R. These articles explore various aspects such as structural studies, analysis of frequent coding polymorphisms, and physiological evaluations, indicating the considerable interest in P2X7R. Molecular determinants of antagonist potency in the allosteric binding pocket of human P2X4R were identified (Pasqualetto et al.). Therefore, through a combination of molecular docking, mutagenesis, and functional assay, they demonstrate the likely binding pocket for the allosteric antagonist BX430, a potent allosteric P2X4R receptor antagonist, to human P2X4R. Interestingly, the importance of the amino acid residue Ile312 for the receptor sensitivity to BX430 is confirmed. These findings have immense potential to aid in the design and development of potent allosteric P2X4R antagonists. In an associated article, the same group identified a novel P2X7R antagonist using structure-based virtual screening (Pasqualetto et al.). This study conducted virtual screening using a molecular model of human P2X4R based on the crystal structure of *Danio rerio* P2X4R. From a library of 300,000 drug-like compounds, none of these compounds displayed a significant antagonist effect in P2X4R. However, when the same set of compounds was tested against human P2X7, one compound (GP-25) showed partial antagonistic activity. Next, in a separate article on the Research Topic, effective antagonism of microglial P2X7R is accomplished using nanobodies and nanobody-encoding adeno-associated virus (Pinto-Espinoza et al.). The results obtained in this study offer novel and in-depth information on optimal conditions, including route, dose, and administration time, for effective nanobody-mediated receptor targeting of microglia. Thus, the potential of nanobodies as a promising new therapeutic strategy for the treatment of sterile brain inflammation is highlighted. The existence of P2X7Rs in CNS neurones is a matter of ongoing debate. While some argue that only non-neuronal cells bear this receptor type and indirectly signal to neighbouring neurons, others propose that CNS neurons themselves possess these receptors. Additional aspects of this hypothesis are explored (Zhang et al.). Using genetic deletion of P2X7Rs specifically in astrocytes, oligodendrocytes, and microglia, and then recording current responses in neurons to 2ʹ(3ʹ)-O-(4-benzoylbenzoyl)-ATP (Bz-ATP), a P2X7R agonist, the authors demonstrate that pyramidal neurons of mouse CA1 and CA3 did not possess P2X7Rs, but were indirectly modulated by astrocytic and oligodendrocytic P2X7Rs, respectively. Finally, the origin, distribution, and function of three frequent coding polymorphisms in the gene for the human P2X7R ion channel were evaluated (Schäfer et al.). The human P2RX7R gene has multiple single nucleotide polymorphisms (SNPs) but unlike other P2XR family members, non-synonymous SNPs in P2X7R are prevalent. Three of these SNPs have a frequency of more than 25% and impact the extracellular head domain of P2X7R (155 Y/H), the lower body (270 R/H), and the tail in the second transmembrane domain (348 T/A). The authors contribute to a deeper understanding of how the structure of P2X7R influences receptor function and highlight the importance of incorporating P2X7R variants into the design of clinical trials targeting this receptor.

There are eight known subtypes of P2YR, each of which has a different ligand specificity and signalling pathway. The potential role of P2Y_2_R in the cardioprotective effects of transplanted undifferentiated cardiac adipose derived stem cells (cADSC) was examined in a mouse model of myocardial infarction (Diaz Villamil et al.). Their findings suggest that P2Y_2_R serves as a crucial regulator of the therapeutic use of undifferentiated cADSC for the treatment of cardiac ischemia. This new insight could help optimise the development of cardiac repair cell therapies, potentially improving patient outcomes. In a separate article, the potential association between P2Y_4_R mutations and the severity of coronary artery disease (CAD) is studied in a population study involving 50 patients diagnosed with (CAD) and 50 age-matched control individuals (Horckmans et al.). Accordingly, the authors focus on a polymorphism in the coding region (rs3745601) that results in the replacement of asparagine at residue 178 with threonine (N178T) located in the P2Y_4_R second extracellular loop. The N178T variant represents a loss-of-function of the receptor that is associated with less severe coronary artery atherosclerosis and lower fasting plasma glucose in coronary patients.

Another article investigated the impact of bisphosphonate clodronate, which has been suggested to act as a potent vesicular nucleotide transporter (VNUT) blocker *in vitro*, on the spontaneous and/or electrically evoked release of ATP/metabolites and noradrenaline from the perivascular nerve terminals in the mesentery sympathetic nervous system (Donoso et al.). Surprisingly, the authors demonstrate that clodronate inhibits adenosine deaminase activity in isolated endothelial cells as in a crude extract preparation, a finding that may explain the accumulation of adenosine after clodronate mesentery perfusion. Subsequently, the role of A_2A_R signalling in sudden unexpected death in epilepsy (SUDEP) is evaluated (Shen et al.). The article introduces a novel animal model of SUDEP using a combined paradigm of intrahippocampal and intraperitoneal administration of kainic acid (KA) in mice with genetically modified adenosine kinase (ADK) knockdown (Adk^+/−^), which exhibit reduced ADK in the brain. Through this model, the authors identified a critical role for A_2A_R in the nucleus tractus solitarius in the pathophysiology of SUDEP, highlighting A_2A_R as a potential therapeutic target to prevent the risk of SUDEP. Guanine-based purines (GBPs) exert numerous biological effects in the central nervous system through putative membrane receptors, the existence of which is still elusive. To shed light on this question, Garozzo et al. conducted a screening of orphan and poorly characterized G protein-coupled receptors (GPRs) expressed in glioma cells, where GBPs showed an antiproliferative effect. Among the GPRs tested, GPR23, also known as the lysophosphatidic acid 4 receptor, was found to counteract the growth inhibition caused by GBPs in U87 cells (Garozzo et al.). These data indicate the involvement of GPR23 in modulating guanine responses in tumour cell lines.

In general, this Research Topic explores in detail the wide-ranging physiological functions of purines and the underlying structural and mechanistic basis of purinergic signalling. It holds significant promise for the development of innovative therapies for both chronic and acute diseases, while posing a challenge to achieving drug selectivity. The potential to translate basic purinergic knowledge into clinical opportunities is enormous, and further exploration of this field could lead to exciting breakthroughs in pharmacotherapy.

